# Introduction to the April 2026 Issue

**DOI:** 10.1017/psy.2026.10113

**Published:** 2026-05-18

**Authors:** Sandip Sinharay

**Affiliations:** ETS Research Institute, USA Email: ssinharay@ets.org

Dear *Psychometrika* Readers,

Welcome to the second Psychometrika issue of 2026. By now, you should have heard about your proposal to IMPS2026 to be held in Seoul, Korea. I am hoping to meet many of you during the meeting.

This issue first includes thirteen “Theory and Methods” section articles. In the first of these, Xiuxiu Tang and Ying Cheng propose a likelihood-based profile shrinkage algorithm to simplify the item selection process and reduce the computational cost in cognitive diagnostic computerized adaptive testing. The second, by Youmi Suk and Chan Park, proposes a novel causal mediation framework for identifying and estimating functional natural direct effects, functional natural indirect effects, and functional total effects, along with subgroup effects of educational programs on outcomes. In the third, Max Welz proposes for the polyserial correlation model a novel estimator that is designed to be robust against the adverse effects of observations discrepant to the model. In the fourth, Stephen Broomell, Sabina Sloman, and Lisheng He propose a new methodological framework for understanding the generalizability of behavioral modeling results using multivariate sampling distributions for the model parameters. A set of three articles on factor analysis follow. In the first of these, Philipp Sterzinger, Ioannis Kosmidis, and Irini Moustaki introduce a novel maximum softly penalized likelihood framework for factor analysis models to address improper solutions known as Heywood cases that frequently occur in statistical practice; in the second of these, Edgar Merkle, Sonja Winter and, Ellen Fitzsimmons consider how identification constraints in ordinal factor analysis can mimic the treatment of ordinal variables as continuous; the third of these, by Nils Sturma, Miriam Kranzlmueller, Irem Portakal, and Mathias Drton, connects sparse confirmatory factor analysis models to bipartite graphs and provides sufficient graphical conditions for identifiability of the factor loading matrix. The eighth article of this issue, by Zhifei Li and Hongbo Wen, combines the structural equation likelihood function (SELF) method with a recursive partitioning method to achieve an interpretable model of multivariate causal direction heterogeneity in multivariable settings. Two articles focusing on item response theory follow. In the first of these articles, Wenjie Zhou and Lei Guo introduce the multiple response model with inter-option local dependencies, and its simplified version, the multiple response model, for multiple response items such as multiple true–false, multiple-select, and select-*N* items; the second of these, by Seewoo Li and Guemin Lee, proposes a new approach to estimating the latent distribution of item response theory using kernel density estimation. The eleventh article, by Selena Wang, Tracy Sweet, and Subhadeep Paul, attempts to understand the covarying ties between network and item responses using a novel joint model with correlated latent variables. In the twelfth article, Xue Wang, Yinghan Chen, and Shiyu Wang propose a novel Bayesian estimation method that simultaneously learns the Q-matrix, the attribute hierarchy, and the parameters of the deterministic inputs, noisy “and” gate model. The thirteenth article, by Zhongyuan Lyu and Yuqi Gu, proposes a novel two-stage algorithm for latent class models suited for high-dimensional binary responses.

This Psychometrika issue ends with an erratum written by Andrea Brancaccio and coauthors of their article published in Psychometrika in 2025.

Hope you enjoy the issue.

